# Identifying High‐Risk Children Safe for Same‐Day Discharge After Tonsillectomy

**DOI:** 10.1002/lary.70629

**Published:** 2026-05-14

**Authors:** Amy Ho, Yann‐Fuu Kou, Christopher Liu, Cynthia S. Wang, Felicity Lenes‐Voit, Stephen R. Chorney, Kenneth Lee, Seckin Ulualp, Ron B. Mitchell, Romaine F. Johnson

**Affiliations:** ^1^ Department of Otolaryngology—Head and Neck Surgery University of Texas Southwestern Medical Center Dallas Texas USA; ^2^ Division of Pediatric Otolaryngology Children's Health Dallas Dallas Texas USA

**Keywords:** Bayesian analysis, obstructive sleep apnea, pediatric obesity, polysomnography, postoperative complications, risk assessment, T&A outcomes

## Abstract

**Objective:**

Current guidelines recommend overnight admission for children with severe obstructive sleep apnea (OSA) and obesity undergoing tonsillectomy, although most have uneventful postoperative courses. We aimed to identify low‐risk subgroups within this high‐risk population who may be candidates for same‐day discharge.

**Methods:**

Retrospective cohort study of children aged 2–18 years with obesity (≥ 95th BMI percentile) and severe OSA (AHI ≥ 10 events/h and/or SpO_2_ nadir < 80%) undergoing tonsillectomy at a tertiary children's hospital (2021–2024). The primary outcome was a severe perioperative event: ICU admission, prolonged hospitalization (> 48 h), or need for advanced respiratory support. Bayesian logistic regression using informative priors identified predictors. Model‐based risk stratification and simplified clinical rules were evaluated using sensitivity, specificity, and negative predictive value.

**Results:**

Among 304 children, 36 (11.8%) experienced a severe event. SpO_2_ nadir was the strongest predictor (*β* = −0.668, 95% CrI [−1.164, −0.172], directional probability 99.6%), followed by Class III obesity (97.0%) and AHI (95.0%). Model‐derived stratification identified 33% of patients with < 5% predicted probability of severe events. A clinical rule combining AHI < 25 events/h and SpO_2_ nadir > 85% showed sensitivity 85.7% and negative predictive value 99.5%, corresponding to an observed event rate < 5%.

**Conclusion:**

Most obese children with severe OSA do not experience severe perioperative events. Simple polysomnographic criteria can identify very low‐risk patients who may be candidates for same‐day discharge planning. These findings support a more individualized approach to postoperative admission; prospective validation is needed before implementation.

**Level of Evidence:**

4.

## 1 Introduction

Over 500,000 tonsillectomies occur annually in the United States, with 5%–6% experiencing postoperative complications. Despite the low risk of complications, the American Academy of Otolaryngology—Head and Neck Surgery (AAO‐HNS) tonsillectomy clinical practice guidelines (CPGs) recommend postoperative overnight admission for high‐risk patients; for example, children with severe obstructive sleep apnea (OSA) and severe obesity (BMI% > 120th of the 95th) [1]. While this approach prioritizes safety, it treats a clinically heterogeneous population as uniformly high risk.

In practice, most children meeting these criteria have uneventful postoperative courses. However, routine admission of all high‐risk patients carries substantial cost burden [2] at roughly $331 million per year and creates a bottleneck in operating room efficiency [3]. This means reserving inpatient beds for children who largely will not need them, limiting surgical capacity to a fraction of what could otherwise be achieved. The result is longer wait times for families and delayed treatment for a condition with known developmental consequences. These considerations raise a key question: can we better distinguish which patients within this high‐risk population truly require admission?

Previous studies have identified risk factors for postoperative complications, with polysomnogram (PSG) parameters—particularly measures of oxygen desaturation such as SpO_2_ nadir and time spent below 90% saturation (TB90)—emerging as stronger predictors than the apnea‐hypopnea index (AHI) or body mass index (BMI) percentile alone [4, 5, 6]. However, these studies have focused on identifying predictors of complications rather than determining which patients can be safely managed without overnight observation. Of note, severe complications occur in only 12.5% to 17% of cases, suggesting that the majority of patients classified as high risk do not experience clinically significant events. Therefore, current CPGs may inadvertently obscure clinically relevant variation, resulting in unnecessary healthcare utilization.

The primary aim of this study was to examine obese children with severe OSA undergoing tonsillectomy with or without adenoidectomy to identify subgroups at lower risk of severe perioperative complications. We hypothesize that select patients with more favorable PSG parameters could be safely considered for same‐day discharge planning, while maintaining admission for those with higher risk or clinical indication. Notably, the AAO‐HNSF CPG identified as a research need the development of validated severity scales for polysomnography to inform hospital admission and perioperative monitoring in children with severe OSA [1]. This study directly addresses that need.

## 2 Methods

This study was approved by the UT Southwestern Medical Center Institutional Review Board (STU# 2023‐0260) as exempt from requiring consent due to the retrospective study design and minimal risk of harm.

### 2.1 Study Population

We retrospectively studied children aged 2–18 years who underwent tonsillectomy between January 2021 and January 2024 at a tertiary care academic children's hospital using data from the electronic medical record. Inclusion criteria were: (1) obesity (≥ 95th BMI percentile) and (2) severe OSA (AHI ≥ 10 events/h and/or SpO_2_ nadir < 80%) confirmed by preoperative polysomnography. We excluded patients with tracheostomy, Down syndrome, mucopolysaccharidosis, neuromuscular disorders, and craniofacial abnormalities.

### 2.2 Surgical Technique

Tonsillectomy was performed by 12 attending surgeons with resident involvement; surgical techniques varied across providers and included electrocautery and coblation approaches. We did not stratify by technique because this study examines the safety of discharge disposition rather than the comparative effectiveness of surgical approaches, and current AAO‐HNS guidelines do not condition admission recommendations on surgical technique.

### 2.3 Outcome Measures

The primary outcome was a composite of perioperative complications (hereafter referred to as a severe event), as defined by Kou et al.: ICU admission, length of stay > 48 h, and the need for respiratory support (high flow nasal cannula [HFNC] > 5 L/min, continuous positive airway pressure or bilevel positive airway pressure [CPAP/BiPAP], or endotracheal intubation). Because prolonged length of stay may reflect both clinical severity and institutional practice patterns, we performed a sensitivity analysis using a respiratory‐focused composite outcome (ICU admission or need for respiratory support).

Secondary outcomes included documented bronchospasm, laryngospasm, hypoxia, oxygen therapy, 30‐day emergency department (ED) visits or readmissions, and returns for bleeding or severe pain/dehydration. Return visits were defined based on the chief complaint and discharge summary of the ED visit. If the family cited bleeding as the reason for the revisit, it was counted as a return for bleeding even if bleeding was not observed on physical exam.

### 2.4 Data Collection

We collected demographic information (age, sex, self‐identified race, insurance status), comorbidities (asthma, gastroesophageal reflux disease (GERD), allergic rhinitis, prematurity, hearing loss, allergies), polysomnography parameters, and anthropometric measurements. Comorbidities were determined from the patient's active problem list within the electronic medical record. BMI classification followed the 2022 Centers for Disease Control (CDC) extended BMI‐for‐Age growth charts, categorizing obesity as Class I (95%–120% of the 95th percentile), Class II (120%–140% of the 95th percentile or BMI 35–40), and Class III (≥ 140% of the 95th percentile or BMI ≥ 40). Perioperative data included tonsillar size and the total length of stay.

### 2.5 Statistical Analysis

To address potential bias, we created a Directed Acyclic Graph (DAG) to map causal pathways during the pre‐writing phase to identify exposures, mediators, outcomes, and confounders (see Figure S1).

We employed Bayesian logistic regression to assess associations between a severe event and key predictors identified from previous studies: age, Black race, obesity classification, and polysomnographic parameters (AHI, SpO_2_ nadir, TB90, and peak end‐tidal CO_2_). Continuous variables were standardized (mean = 0, SD = 1) before inclusion in the model. We employed standardized measures to improve direct comparisons between variables and model stability.

Informative priors based on studies by Lim and Kou were specified to incorporate existing evidence (see Tables S1 and S2 for exact priors). We estimated the posterior distribution using four independent Markov Chain Monte Carlo chains of 50,000 iterations each, with 10,000 iterations discarded as burn‐in and a thinning rate of 5. Model convergence was assessed using diagnostic plot visualization and Gelman–Rubin diagnostics (values < 1.1 considered adequate).

Based on model‐identified predictors, we evaluated simplified clinical decision rules using clinically interpretable thresholds for AHI and SpO_2_ nadir. We used the model to predict each patient's probability of experiencing a severe event, stratifying subjects into three risk groups: Group 1 (≤ 2.5% probability), Group 2 (≤ 5% probability), and Group 3 (≤ 10% probability). We compared these model‐derived groups to clinically defined lower‐risk among high‐risk criteria (AHI < 20 events/h and SpO_2_ nadir > 85), calculating sensitivity, specificity, positive and negative predictive value for each probability threshold. Sensitivity analyses evaluated alternative clinical criteria to test the robustness of the model, including a more restrictive definition (AHI ≤ 15 events/h) and a more liberal one (AHI ≤ 25 events/h), while maintaining the SpO_2_ nadir threshold at > 85%.

Continuous variables are presented as means with standard deviation (SD) or medians with interquartile ranges (IQR) when indicated. Categorical variables are presented as counts with percentages. Bayesian results are presented as posterior means with 95% credible intervals (CrI); regression tables include coefficient means, SD, Monte Carlo Standard Error (MCSE), medians, 95% CrI, and Gelman–Rubin statistic (R‐hat). In addition to posterior means and CrI, we calculated directional posterior probabilities to assess evidence strength for each parameter. The probability (*p*) that (*β* > 0 or *β* < 0) was considered strong (*p* > 95%), moderate (95% > *p* > 75%), and weak (*p* < 75%).

Power analysis indicated 282 patients were required to detect a difference between a 15% baseline outcome rate and 5% in the low‐risk population (80% power, *α* = 0.05). Analyses were performed using Stata Statistical Software version 19 (College Station, TX). Missing data was minimal (< 2%) and assumed to be missing at random; therefore, complete case analysis was performed. This study adhered to Strengthening the Reporting of Observational studies in Epidemiology (STROBE) reporting guidelines.

## 3 Results

### 3.1 Study Population

Of the 304 children meeting inclusion criteria (*N* = 304), 36 (11.8%) experienced a severe event. For the cohort, the median (IQR) age was 8 years (6.0), 181 (59.5%) were male, and 203 (66.8%) were Hispanic. The majority had Class I obesity (*N* = 126, 41.4%), followed by Class II (*N* = 108, 35.5%) and Class III (*N* = 70, 23.0%). Comorbidities included asthma (*N* = 72, 23.7%), allergic rhinitis (*N* = 52, 17.1%), history of premature birth (*N* = 24, 7.9%), GERD (*N* = 12, 3.9%), and congenital heart disease (*N* = 7, 2.3%). Children who experienced severe events tended to be more obese, with 41.7% having Class III obesity compared to 20.5% in the non‐severe event group. See Table 1 for complete demographic characteristics.

**TABLE 1:** Demographics by severe event among obese children undergoing tonsillectomy.

	No severe event 268 (88.2%)	Severe event 36 (11.8%)	Total 304 (100%)
Age at surgery	8.5 (6.0)	8 (8.0)	8 (6.0)
Height (cm)	141 (35.0)	135 (40.0)	140 (36.0)
Weight (kg)	57 (43.0)	62 (69.0)	57 (46.0)
BMI	28 (9.6)	32 (14.0)	28 (10.0)
BMI percentile	129 (31.0)	144 (39.0)	131 (33.0)
BMI *Z* score	2.5 (1.0)	3.4 (1.5)	2.6 (1.2)
Obesity classification			
Class I	118 (44.0%)	8 (22.2%)	126 (41.4%)
Class II (severe)	95 (35.4%)	13 (36.1%)	108 (35.5%)
Class III (very severe)	55 (20.5%)	15 (41.7%)	70 (23.0%)
Sex			
Male	157 (58.6%)	24 (66.7%)	181 (59.5%)
Female	111 (41.4%)	12 (33.3%)	123 (40.5%)
Race			
White or Caucasian	159 (59.3%)	20 (55.6%)	179 (58.9%)
Black or African American	63 (23.5%)	12 (33.3%)	75 (24.7%)
Asian	7 (2.6%)	0 (0.0%)	7 (2.3%)
Native American/Pacific Islander	2 (0.7%)	0 (0.0%)	2 (0.7%)
Other/Unknown	37 (13.8%)	4 (11.1%)	41 (13.5%)
Ethnicity			
Not Hispanic	89 (33.2%)	12 (33.3%)	101 (33.2%)
Hispanic	179 (66.8%)	24 (66.7%)	203 (66.8%)
Asthma	63 (23.5%)	9 (25.0%)	72 (23.7%)
GERD	11 (4.1%)	1 (2.8%)	12 (3.9%)
Allergic rhinitis	48 (17.9%)	4 (11.1%)	52 (17.1%)
Premature birth	18 (6.7%)	6 (16.7%)	24 (7.9%)
Allergies	16 (6.0%)	2 (5.6%)	18 (5.9%)
Congenital heart disease	5 (1.9%)	2 (5.6%)	7 (2.3%)

*Note:* Continuous variables are median (IQR). Categorical variables are *N* (%). Obesity in children is classified relative to the sex‐ and age‐specific 95th BMI percentile (BMI_95_) on CDC growth charts. Class I obesity: BMI ≥ 95th percentile to < 120% of BMI_95_. Class II obesity: BMI ≥ 120% to < 140% of BMI_95_, or BMI ≥ 35 kg/m^2^, whichever is lower. Class III obesity: BMI ≥ 140% of BMI_95_, or BMI ≥ 40 kg/m^2^, whichever is lower.

### 3.2 Outcome Measures

Among the 36 patients with severe events, 20 (55.6%) required ICU admission, 17 (47.2%) required advanced respiratory support, and 32 (88.9%) had length of stay exceeding 48 h. Secondary outcomes differed between groups: post‐tonsillectomy bleeding occurred in 16.7% of the severe event group versus 7.1% in the non‐severe event group. ED visits within 30 days were more common in the severe event group (25.0% vs. 17.9%), as were readmissions (8.3% vs. 6.7%). See Table 2 for complete perioperative outcomes.

**TABLE 2:** Perioperative outcomes by severe event.

	No severe event 268 (88.2%)	Severe event 36 (11.8%)	Total 304 (100%)
Length of stay (days)	1 (0)	2 (2.5)	1 (0)
ICU admission	0 (0%)	20 (55.6%)	20 (6.6%)
Dexamethasone	260 (97.0%)	34 (94.4%)	294 (96.7%)
Laryngospasm	1 (0.4%)	1 (2.8%)	2 (0.7%)
Bronchospasm	2 (0.7%)	1 (2.8%)	3 (1.0%)
Hypoxemia	17 (6.3%)	4 (11.4%)	21 (6.9%)
Oxygen therapy	0 (0%)	29 (80.6%)	29 (9.5%)
Respiratory support	0 (0%)	17 (47.2%)	17 (5.6%)
Post‐tonsillectomy bleeding	19 (7.1%)	6 (16.7%)	25 (8.2%)
Severe pain or dehydration	25 (9.4%)	6 (16.7%)	31 (10.2%)
ED visit	48 (17.9%)	9 (25.0%)	57 (18.8%)
Readmission	18 (6.7%)	3 (8.3%)	21 (6.9%)

*Note:* Continuous variables are median (IQR). Categorical variables are *N* (%).

### 3.3 Polysomnography Findings

Preoperative polysomnographic findings are presented in Table 3 (*N* = 304). All patients had severe OSA (AHI ≥ 10 events/h), and 124 (40.8%) had very severe OSA (AHI > 30 events/h). For the entire cohort, the median (IQR) AHI was 24.3 events/h (31.0), SpO_2_ nadir was 83% (12.0), TB90 was 2.8% (11.1), and peak CO_2_ was 51 mmHg (6.5). Patients with severe events demonstrated consistently worse PSG measurements: AHI was nearly twice as high (41.1 vs. 22.8 events/h), SpO_2_ nadir was substantially lower (69.5% vs. 84.0%), and TB90 was markedly elevated (18.6% vs. 2.4%).

**TABLE 3:** Preoperative polysomnography by severe event.

	No severe event 268 (88.2%)	Severe event 36 (11.8%)	Total 304 (100%)
Age at PSG	8.69 (6.3)	8.00 (7.4)	8.61 (6.4)
Total sleep time	397.50 (118.5)	208.50 (268.5)	391.00 (139.0)
Sleep efficiency	80.50 (16.9)	75.25 (23.5)	79.80 (17.4)
Sleep latency	30.00 (47.0)	40.00 (58.0)	30.50 (47.0)
REM latency	124.00 (112.0)	90.50 (101.0)	119.00 (107.0)
N1	17.00 (15.5)	12.00 (15.5)	17.00 (16.0)
N2	196.50 (77.5)	103.00 (124.0)	192.50 (90.0)
N3	93.00 (47.8)	64.50 (82.2)	91.75 (54.5)
NREM	317.50 (87.8)	171.25 (206.8)	313.25 (101.2)
REM	65.50 (54.5)	34.50 (60.0)	64.00 (55.0)
Arousal	112.00 (85.5)	112.50 (102.5)	112.00 (88.5)
OA	37.00 (71.0)	47.50 (75.0)	39.00 (74.5)
OH	88.00 (78.0)	93.50 (85.5)	88.00 (79.0)
oAHI	22.10 (28.5)	42.40 (84.3)	23.30 (30.5)
CAI	0.30 (1.0)	0.30 (2.4)	0.30 (1.2)
AHI	22.80 (28.4)	41.10 (87.0)	24.30 (31.0)
TB90 (% TST < 90%)	2.40 (8.8)	18.55 (62.0)	2.80 (11.1)
SpO_2_ Nadir	84.00 (10.5)	69.50 (23.5)	83.00 (12.0)
Mean SpO_2_	97.00 (2.0)	95.50 (5.5)	97.00 (2.0)
Peak CO_2_	51.00 (6.5)	54.00 (10.0)	51.00 (6.5)
Average CO_2_	44.00 (6.0)	44.00 (9.0)	44.00 (6.0)
% TST > 50 mmHg CO_2_	0.10 (2.8)	1.95 (15.6)	0.20 (3.2)
Total # of PLMS	0.00 (6.0)	0.00 (0.0)	0.00 (4.5)
Very severe OSA (AHI > 30)	101 (37.7%)	23 (63.9%)	124 (40.8%)

*Note:* Continuous variables are median (IQR). TB90 = percentage of total sleep time with SpO_2_ < 90%.

### 3.4 Statistical Analysis

Bayesian modeling was performed including and excluding Black race as a covariate (*N* = 304). The model including race demonstrated high correlation and poor mixing, and AIC testing confirmed race was not needed for optimal model fit; therefore, it was excluded. The final model is shown in Table 4, with complete diagnostics in Tables 1 and 2 and trace graphs in Figures S2 and S3.

**TABLE 4:** Bayesian regression model for severe event among obese children with severe OSA.

Parameter	Posterior mean	SD	95% CrI	Directional probability	Evidence strength
SpO_2_ nadir (std)	−0.668	0.253	−1.164, −0.172	99.55%	Strong
Class III Obesity	0.678	0.361	−0.031, 1.383	96.97%	Strong
AHI (std)	0.307	0.187	−0.059, 0.674	95.02%	Strong
Class II Obesity	0.371	0.339	−0.297, 1.032	86.41%	Moderate
Peak CO_2_ (std)	0.208	0.186	−0.151, 0.582	87.00%	Moderate
Age (std)	−0.188	0.186	−0.559, 0.169	84.49%	Moderate
Time < 90% (std)	0.132	0.208	−0.277, 0.540	73.91%	Weak

*Note:* Posterior mean: Average parameter value from posterior samples. Directional Probability: *p*(*β* > 0) for risk factors; *p*(*β* < 0) for protective factors. Evidence strength: Strong (> 95%), Moderate (75%–95%), Weak (< 75%).

Abbreviation: 95% CrI: 95% credible interval.

The model identified SpO_2_ nadir as the strongest predictor of severe events, showing a strong negative effect (*β* = −0.668, 95% CrI [−1.164, −0.172]) with 99.55% directional probability. Class III obesity was the second strongest predictor (*β* = 0.678, 95% CrI [−0.031, 1.383], directional probability 96.97%, OR 1.97). AHI showed strong evidence of association (*β* = 0.307, 95% CrI [−0.059, 0.674], directional probability 95.02%, OR 1.36). Moderate associations were observed for Class II obesity, peak CO_2_, and age. Notably, TB90 was the weakest predictor (directional probability 73.91%).

The model‐predicted mean probability of severe events was 0.12 (range 0.02–0.78). Based on model‐derived predicted probabilities, 7 patients (2.3%) were classified as ≤ 2.5% risk, 101 (33.2%) as ≤ 5% risk, and 211 (69.4%) as ≤ 10% risk. Thus, when incorporating the strongest predictors, a third of this high‐risk population had < 5% probability of severe events.

Clinical rule performance is shown in Table 5. For identifying patients with ≤ 2.5% probability of complications, AHI < 25 events/h and SpO_2_ nadir > 85% demonstrated the highest sensitivity (85.7%) and NPV (99.5%) while maintaining adequate specificity (72.7%). Among patients meeting these criteria, the observed rate of severe events was low (< 5%).

**TABLE 5:** Performance of clinical rules for identifying low‐risk patients.

Clinical rule	Sensitivity	Specificity	NPV	PPV	OR
For ≤ 2.5% probability (very low risk)					
AHI ≤ 15 and SpO_2_ > 85	71.4%	84.8%	99.2%	10.0%	14.0
AHI < 20 and SpO_2_ > 85	71.4%	74.1%	99.1%	6.1%	7.14
AHI < 25 and SpO_2_ > 85	85.7%	72.7%	99.5%	6.9%	16.0
For ≤ 5.0% probability (low risk)					
AHI ≤ 15 and SpO_2_ > 85	35.6%	93.1%	74.4%	72.0%	7.48
AHI < 20 and SpO_2_ > 85	57.4%	88.2%	80.6%	70.7%	10.1
AHI < 25 and SpO_2_ > 85	63.4%	88.7%	82.9%	73.6%	13.5

Abbreviations: AHI = apnea hypopnea index; NPV = negative predictive value; OR = odds ratio; PPV = positive predictive value; SpO_2_ = blood oxygen saturation nadir.

At the ≤ 5.0% threshold, trade‐offs were observed: AHI ≤ 15 events/h demonstrated higher specificity (93.1%) but lower sensitivity (35.6%), while AHI < 25 events/h provided higher sensitivity (63.4%) with similar specificity (88.7%). All rules maintained NPV > 99% at the very low‐risk threshold, suggesting select patients meeting these criteria could be considered for same‐day discharge.

## 4 Discussion

This study of obese children with severe OSA found that 88.2% did not experience severe perioperative tonsillectomy complications. These findings highlight substantial heterogeneity within a population currently classified as uniformly high risk. The Bayesian model identified SpO_2_ nadir as the strongest predictor of severe complications, followed by Class III obesity and AHI. Simple clinical rules (AHI < 25 events/h and SpO_2_ nadir > 85%) demonstrated excellent performance for identifying very low‐risk patients within this high‐risk population. Importantly, patients meeting these criteria had a low observed event rate (< 5%), supporting the clinical relevance of this stratification approach. The clinical implication is that, rather than reserving inpatient beds for all high‐risk patients, a subset may be considered for same‐day discharge planning—with the understanding that admission remains available based on clinical status. This approach does not lower the threshold for admission; it expands the pool of patients who can be scheduled without guaranteed bed reservation.

Same‐day discharge in this context refers to discharge from the post‐anesthesia care unit following standard recovery, not extended 23‐h observation, which represents current practice for this high‐risk population. The distinction is clinically important: extended observation models maintain overnight resource utilization, whereas our criteria identify patients for whom standard PACU recovery and same‐day discharge could be the default plan.

SpO_2_ nadir emerged as the strongest predictor of severe perioperative complications—stronger than AHI. This finding aligns with accumulating evidence that nocturnal oxygen desaturation carries greater physiologic consequence than the frequency of apneas and hypopneas alone. Current guidelines include SpO_2_ nadir < 80% in defining severe OSA, and prior literature consistently identifies SpO_2_ nadir as a predictor of postoperative respiratory events [1, 5, 6, 7]. The SpO_2_ nadir > 85% threshold identified by our model represents a conservative boundary: still abnormal, but not critically so. We deliberately avoided pushing this threshold lower because the data genuinely support caution with more severe desaturations.

Class III obesity was the second strongest predictor. Prior studies have identified obesity as a risk factor for severe OSA with higher probability of residual OSA post‐tonsillectomy [8], and obese children have higher respiratory complications compared to non‐obese children [9, 10]. In our model, obesity likely reflects underlying physiologic risk mediated through disordered ventilation and oxygenation, rather than acting as an independent driver of complications. Our study further stratifies risk by incorporating three obesity classes, demonstrating that Class I obesity was not a significant predictor. Notably, one study found no association between obesity severity and respiratory events [11]; however, that study evaluated only two categories (≥ 95% and ≥ 120%), which differed from our classification. The importance of Class III obesity also underscores the necessity of polysomnography in this population: clinical features alone cannot adequately characterize the degree of sleep derangement or perioperative risk in severely obese children.

We deliberately excluded Class III obesity from the simple clinical rule for two reasons. First, our prior work demonstrates that respiratory complications are primarily mediated through obstructive and respiratory pathways; SpO_2_ nadir and AHI directly measure these pathways, whereas obesity class is a proxy that confers risk largely through its effects on nocturnal oxygenation. Including obesity class alongside PSG values risks double‐counting the same underlying physiologic risk. Second, a two‐variable rule based on familiar metrics is immediately implementable in existing clinical workflows; adding a third criterion moves toward a risk calculator, which is premature without prospective validation.

AHI, a classic criterion for defining severe OSA, was the third strongest predictor. Some may argue that thresholds like AHI < 25 events/h and SpO_2_ nadir > 85% are arbitrary, but existing cutoffs are similarly derived. The widely used AHI > 10 events/h derives from small cohort studies measuring SDs from a reference population, not from outcome data. The AAP uses AHI > 24 events/h, reflecting uncertainty about where meaningful risk begins [12, 13]. Our thresholds are empirically derived from Bayesian modeling incorporating prior studies and calibrated to existing clinical workflows. We already think in terms of AHI and SpO_2_ nadir; therefore, this approach refines those familiar metrics rather than introducing novel variables.

Our model found weak association between TB90 and severe events (directional probability 73.91%). This difference may reflect the distinction between predicting any respiratory event and identifying patients safe for discharge, where threshold‐based physiologic extremes (e.g., nadir oxygenation) may better discriminate low‐risk patients. This contrasts with prior studies identifying TB90 as among the strongest predictors of severe respiratory events [5, 7, 14]. Our focus on modeling safe discharge rather than identifying respiratory event risk may explain this difference. Additionally, Bayesian incorporation of prior knowledge may have compressed TB90's effect relative to frequentist approaches used in prior studies.

This study highlights potential reduction in unnecessary overnight admissions for patients currently classified as high‐risk. Rather than redefining high‐risk criteria, this approach refines risk within an already defined high‐risk group, allowing more efficient allocation of inpatient resources and improved surgical access. The economic consideration does not compromise safety; our Bayesian model utilized stratified risk groups with evidence‐based selection criteria. A prior study testing age < 3 years, SpO_2_ nadir, and peak CO_2_ achieved AUC = 0.72, outperforming existing guidelines [7]. Similarly, our parameters (AHI < 25 events/h and SpO_2_ nadir > 85%) could be incorporated into existing workflows for patients already considered high‐risk due to severe OSA and obesity.

Two sensitivity analyses confirmed robustness. First, excluding children younger than 3 years old (*N* = 297) did not materially alter model estimates: SpO_2_ nadir remained the strongest predictor (*β* = −0.564, directional probability 98.4%), and Class III obesity (*β* = 0.689, directional probability 96.9%) and AHI (*β* = 0.308, directional probability 95.3%) were virtually unchanged. Second, to address the concern that length of stay > 48 h may capture non‐respiratory events, we evaluated a respiratory‐focused composite outcome defined as ICU admission or need for respiratory support, excluding LOS > 48 h. Of 304 patients, 23 (7.6%) experienced a respiratory‐focused severe event. Among 87 patients meeting the clinical rule (AHI < 25 events/h and SpO_2_ nadir > 85%), only 1 (1.1%) experienced a respiratory‐focused event, confirming that the clinical rule's performance is not inflated by non‐respiratory prolonged stays.

Strengths include Bayesian analysis incorporating prior knowledge from our group's previous work for improved probability estimation, comprehensive data with near‐complete sleep study parameters and operative events, and 30‐day follow‐up capturing ED visits and readmissions. The downstream consequences observed in Table 3—higher bleeding rates, more ED visits, more readmissions—reinforce why identifying low‐risk patients matters: it is not just about the index admission, but about the broader burden these events place on families and the healthcare system. The stratified risk approach maintains clinical applicability while the use of informative priors strengthens effect estimates beyond what a frequentist analysis of this sample size could achieve.

This study has several limitations. First, our cohort was predominantly Hispanic (66.8%); application to populations with different baseline comorbidities may underestimate perioperative event risk. Second, high obesity prevalence implies our aggregate results likely overestimate risk compared to non‐obese pediatric populations. Third, the absence of preoperative CPAP data—which can mitigate airway obstruction—may have masked untreated physiology severity, potentially underestimating true risk in severe cases. Finally, these criteria assume polysomnography data are available; whether clinical features alone can risk‐stratify without PSG remains an open question, though current evidence suggests PSG is essential, particularly in Class III obesity.

Additionally, comorbidities were ascertained from the active problem list within the electronic medical record, which may underascertain conditions not consistently documented by providers. Any such underascertainment would bias toward the null, making our estimates conservative. Future studies with larger sample sizes could evaluate whether separating cardiorespiratory from non‐cardiorespiratory comorbidities provides additional predictive value beyond PSG parameters.

We are not recommending practice change based on this retrospective analysis. All patients in this cohort met multiple independent criteria for overnight admission under current guidelines, and all would continue to be admitted under the proposed next step. The evidence pathway proceeds in stages: first, this retrospective analysis identifies candidate criteria warranting further study; second, a prospective observational study would continue admitting all patients under existing protocols while flagging those meeting AHI < 25 events/h and SpO_2_ nadir > 85%, evaluating over 6–12 months whether the severe event rate among flagged patients remains below 5%; third, if prospective data validate these criteria, a quality improvement initiative with appropriate institutional guardrails could evaluate implementation of modified discharge protocols; and fourth, publication at each stage ensures peer review throughout. This staged approach directly addresses the AAO‐HNSF guideline's identified research need for validated PSG‐based severity scales to inform admission decisions [1]. Questions raised by this analysis—including surgical technique, comorbidity subtypes, and age‐specific thresholds—are refinement questions best addressed with prospectively collected data and larger sample sizes. If prospective data show risk exceeding acceptable thresholds, the criteria require refinement before proceeding.

## 5 Conclusion

Due to risk heterogeneity, many patients classified as high‐risk for perioperative complications are, in fact, at low risk. Simple polysomnographic criteria (AHI < 25 events/h and SpO_2_ nadir > 85%) can identify a subset of patients with low observed risk of severe events who may be appropriate candidates for same‐day discharge planning. This approach does not lower the threshold for admission but instead refines patient selection within existing high‐risk categories. Prospective validation is warranted before implementation.

## Funding

The authors have nothing to report.

## Disclosure

Presented as a poster at the Triological Society Combined Sections Meeting, Orlando, Florida, January 20–23, 2025.

## Conflicts of Interest

Stephen R. Chorney is an education consultant for Smith and Nephew. Kenneth Lee is the Chief Medical Officer of Qualio Oto. The other authors declare no conflicts of interest.

## Supporting information


**Table S1:** Informative prior specifications for Bayesian logistic regression model parameters, derived from previously published studies by Lim et al. and Kou et al.
**Table S2:** Summary of prior distributions and hyperparameters used in the Bayesian logistic regression model for predicting severe perioperative events.

**Figure S1:** Directed Acyclic Graph (DAG) of causal pathways for severe perioperative events after tonsillectomy in obese children with severe obstructive sleep apnea. The DAG illustrates hypothesized relationships among exposures, mediators, confounders, and outcomes used to guide variable selection for the Bayesian logistic regression model.

**Figure S2:** Diagnostic plots for intercept parameter from Bayesian logistic regression predicting severe events in pediatric OSA (*N* = 303). Model includes race variable, showing trace, histogram, autocorrelation, and density plots from four MCMC chains.

**Figure S3:** Diagnostic plots for intercept parameter from Bayesian logistic regression predicting severe events in pediatric OSA (*N* = 303). Alternative model excluding race variable, with identical diagnostic assessments for comparison.

## Data Availability

The data that support the findings of this study are available on request from the corresponding author. The data are not publicly available due to privacy or ethical restrictions.
