# Supplementary material for: Identifying High‐Risk Children Safe for Same‐Day Discharge After Tonsillectomy
Source: Laryngoscope. 2026 May 14;136(10):4497–504. doi: 10.1002/lary.70629 (PMC13569659; doi:10.1002/lary.70629)
Supplement: Supplementary file 1 [file LARY-136-4497-s001.docx]

**Supplemental Figure 1.** Directed Acyclic Graph of Causal Pathways for Severe Perioperative Events After Tonsillectomy

**Supplemental Figure 2.** Diagnostic plots for intercept parameter from Bayesian logistic regression predicting severe events in pediatric OSA (N=303). Model includes race variable, showing trace, histogram, autocorrelation, and density plots from four MCMC chains.

**Supplemental Figure 3.** Diagnostic plots for intercept parameter from Bayesian logistic regression predicting severe events in pediatric OSA (N=303). Alternative model excluding race variable, with identical diagnostic assessments for comparison.

**Supplement Table 1: Bayesian Model of Severe Event, Including Race as a Covariable.**

| Parameter | Posterior Mean | 95% CrI | MSCE | ESS | R-hat |
| --- | --- | --- | --- | --- | --- |
| Obesity Class |  |  |  |  |  |
| Class II Obesity | 0.405 | (-0.261, 1.067) | 0.002 | 19255 | 1.0001 |
| Class III Obesity | 0.710 | (-.0005, 1.419) | 0.004 | 6861 | 1.0005 |
| Black Race |  |  |  |  |  |
| Yes | 0.319 | (-0.410, 1.023) | 0.003 | 14536 | 1.0000 |
| Standardized variables |  |  |  |  |  |
| Age | -0.195 | (-0.568, 0.168) | 0.002 | 11,010 | 1.0012 |
| AHI | 0.327 | (-0.041, 0.696) | 0.002 | 8,180 | 1.0005 |
| SpO_2_ | -0.654 | (-1.157, -0.149) | 0.004 | 4,346 | 1.0005 |
| TB90 | 0.126 | (-0.283, 0.529) | 0.003 | 6,099 | 1.0004 |
| Peak CO_2_ | 0.197 | (-0.164, 0.571) | 0.002 | 14,237 | 1.0004 |
| _cons | -2.822 | (-3.441, -2.246) | 0.003 | 10,716 | 1.0004 |

Posterior estimates from Bayesian logistic regression predicting severe events in pediatric sleep apnea patients (N=303). Analysis used 4-chain MCMC with 50,000 samples after 10,000 burn-in iterations. High autocorrelation after 500 lags in at least one chain. Default initial values were used for multiple chains. All parameters meet convergence rule (< 1.1). Efficiency = 0.05291

**Abbreviations:** 95% CrI – 95% Credible Interval; MSCE - Monte Carlo Standard Error; ESS - Effective Sample Size; R-hat - Gelman-Rubin convergence diagnostic; AHI – apnea hypopnea index; SpO_2_ - peripheral oxygen saturation nadir; TB90 – time peripheral oxygen saturation spent < 90% during sleep; Peak CO_2_ – peak CO_2_ in mmHg during sleep.

**Supplement Table 2: Bayesian Model of Severe Event, Excluding Race As a Covariable**.

| Parameter | Posterior Mean | 95% CrI | MSCE | ESS | R-hat |
| --- | --- | --- | --- | --- | --- |
| Obesity Class |  |  |  |  |  |
| Class II Obesity | 0.371 | (-0.297, 1.032) | 0.002 | 23663 | 1.0002 |
| Class III Obesity | 0.678 | (-0.031, 1.383) | 0.003 | 13368 | 1.0002 |
| Standardized variables |  |  |  |  |  |
| Age | -0.188 | (-0.559, 0.169) | 0.002 | 10134 | 1.0007 |
| AHI | 0.307 | (-0.059, 0.674) | 0.001 | 18221 | 1.0001 |
| SpO_2_ | -0.668 | (-1.164, -0.172) | 0.002 | 19088 | 1.0001 |
| TB90 | 0.132 | (-0.277, 0.540) | 0.001 | 22534 | 1.0002 |
| Peak CO_2_ | 0.208 | (-0.151, 0.582) | 0.001 | 17035 | 1.0007 |
| _cons | -2.682 | (-3.257, -2.144) | 0.002 | 25338 | 1.0000 |

Posterior estimates from Bayesian logistic regression predicting severe events in pediatric sleep apnea patients (N=303), alternative model excluding race. Analysis used 4-chain MCMC with 50,000 samples after 10,000 burn-in iterations. High autocorrelation after 500 lags in at least one chain. Default initial values were used for multiple chains. All parameters meet convergence rule (< 1.1). Efficiency = 0.09336

**Abbreviations:** 95% CrI – 95% Credible Interval; MSCE - Monte Carlo Standard Error; ESS - Effective Sample Size; R-hat - Gelman-Rubin convergence diagnostic; AHI – apnea hypopnea index; SpO_2_ - peripheral oxygen saturation nadir; TB90 – time peripheral oxygen saturation spent < 90% during sleep; Peak CO_2_ – peak CO_2_ in mmHg during sleep

**Priors Used for Models:**

- Class II Obesity: normal (0.4, 0.25)
- Class III Obesity: normal (0.6, 0.3)
- Black race: normal (0.4, 0.4) (excluded in alternative model)
- Standardized age: normal (-0.5, 0.25)
- Standardized AHI: normal (0.25, 0.2)
- Standardized SpO_2_: normal (-0.5, 0.25)
- Standardized TB90: normal (0.6, 0.25)
- Standardized CO_2_: normal (0.3, 0.2)
- _cons: normal (-2.0, 1.0)
